# New Appliances & Things Medical

**Published:** 1910-08-27

**Authors:** 


### NEW APPLIANCES & THINGS MEDICAL.

**[We shall be glad to receive at our Office, 28 & 29 Southampton Street, Strand, London, W.C., from the manufacturers, specimens of all new preparations and appliances.]**

### NONAL ALE AND STOUT.

(Nonal Company, Ltd., Fulham, London, S.W.)

We have received samples of these ales and stouts, manufactured by the Nonal Company, Ltd., according to Kuhn's patent, and stated to be non-alcoholic. We doubt whether the ordinary person who is not a connoisseur in drinks will be able to tell the difference between these products—especially the ale—and the ordinary ales and stouts sold over the bar. They possess an agreeable, palatable taste, and certainly rank as excellent malt liquors, with the bitter flavour decidedly apparent. The amount of alcohol they contain is very small, and they do not show any trace of extraneous preservative. Both samples submitted to us compared very favourably with samples of ordinary ale and stout so far as the amount of food material present was concerned, except that the amount of sugar present in the Nonal ale was slightly above that to be found in ordinary ale. Those who object to malt liquors on the score of their alcoholic content will do well to give these productions a thorough trial. For convalescents especially we should imagine Nonal stout to be an excellent drink.

### DIGESTIN.

(Burgoyne, Burbidges & Co., 12 and 16 Coleman Street, London, E.C.)

Digestin is a vegetable enzyme derived from the Okazaki fungus, an aspergillus with a peculiar mycelium which grows in the Far East, and is prepared in the pharmaceutical laboratory of Messrs. Yenjo Shoten, Ltd., at Somei, Sugamo, near Tokio, in Japan. It is a yellowish-white, odourless, practically tasteless hygroscopic powder, the dose of which is, for adults, from 0.1 to 0.3 gram, taken with water immediately after a meal. It is stated to be a very powerful proteid digestant, acting equally well in acid and alkaline media. The samples supplied to us were found to possess marked proteolytic and inverting properties. The invert action was specially marked in a neutral medium when the powder was allowed to act upon dry potato-flour. This digestant appears to us to be well worth a thorough trial, as it is easily taken by children, and is undoubtedly preferable—so far at least as can be judged from test-tube experiments—to other vegetable and animal enzymes on the market. The agents are Messrs. Burgoyne, Burbidges & Co., of 12 and 16 Coleman Street, E.C. The price of the samples was not stated.

